# Vegetation Indices for Early Grey Mould Detection in Lettuce Grown under Different Lighting Conditions

**DOI:** 10.3390/plants12234042

**Published:** 2023-11-30

**Authors:** Asta Kupčinskienė, Aušra Brazaitytė, Neringa Rasiukevičiūtė, Alma Valiuškaitė, Armina Morkeliūnė, Viktorija Vaštakaitė-Kairienė

**Affiliations:** Lithuanian Research Centre for Agriculture and Forestry, Institute of Horticulture, Kaunas Str. 30, LT-54333 Babtai, Lithuania; ausra.brazaityte@lammc.lt (A.B.); neringa.rasiukeviciute@lammc.lt (N.R.); alma.valiuskaite@lammc.lt (A.V.); armina.morkeliune@lammc.lt (A.M.); viktorija.vastakaite-kairiene@lammc.lt (V.V.-K.)

**Keywords:** leafy vegetables, *Botrytis cinerea* Pers., inoculation, HPS, LEDs, non-destructive measurements

## Abstract

Early detection of pathogenic fungi in controlled environment areas can prevent major food production losses. Grey mould caused by *Botrytis cinerea* is often detected as an infection on lettuce. This paper explores the use of vegetation indices for early detection and monitoring of grey mould on lettuce under different lighting conditions in controlled environment chambers. The aim was focused on the potential of using vegetation indices for the early detection of grey mould and on evaluating their changes during disease development in lettuce grown under different lighting conditions. The experiment took place in controlled environment chambers, where day/night temperatures were 21 ± 2/17 ± 2 °C, a 16 h photoperiod was established, and relative humidity was 70 ± 10% under different lighting conditions: high-pressure sodium (HPS) and light-emitting diode (LED) lamps. Lettuces were inoculated by 7-day-old fungus *Botrytis cinerea* isolate at the BBCH 21. As a control, non-inoculated lettuces were grown under HPS and LEDs (non-inoculated). Then, the following were evaluated: Anthocyanin Reflectance Index 2 (ARI2); Carotenoid Reflectance Index 2 (CRI2); Structure Intensive Pigment Index (SIPI); Flavanol Reflectance Index (FRI); Greenness (G); Greenness 2 (G2); Redness (R); Blue (B); Blue Green Index 2 (BGI2); Browning Index 2 (BRI2); Lichtenthaler Index 1 (LIC1); Pigment Specific Simple Ratio (PSSRa and PSSRb); Gitelson and Merzlyak (GM1 and GM2); Zarco Tejada–Miller Index (ZMI); Normalized Difference Vegetation Index (NDVI); Simple Ratio (SR); Red-Eye Vegetation Stress Index (RVSI); Photochemical Reflectance Index (PRI); Photochemical Reflectance Index 515 (PRI515); Water Band Index (WBI); specific disease index for individual study (fD); Healthy Index (HI); Plant Senescence Reflectance (PSRI); Vogelmann Red Edge Index (VREI1); Red Edge Normalized Difference Vegetation Index (RENDVI); and Modified Red Edge Simple Ratio (MRESRI). Our results showed that the PSRI and fD vegetation indices significantly detected grey mould on lettuce grown under both lighting systems (HPS and LEDs) the day after inoculation. The results conclusively affirmed that NDVI, PSRI, HI, fD, WBI, RVSI, PRI, PRI515, CRI2, SIPI, chlorophyll index PSSRb, and coloration index B were identified as the best indicators for *Botrytis cinerea* infection on green-leaf lettuce (*Lactuca sativa* L. cv Little Gem) at the early stage of inoculated lettuce’s antioxidative response against grey mould with a significant increase in chlorophyll indices.

## 1. Introduction

*Botrytis cinerea* (Pers.) Fr., which causes grey mould, is one of the most harmful pathogens because of its wide host range and flexible survival behaviour. Consequently, *B. cinerea* infects host plants such as vegetables, fruits, berries, field canopies, and ornamental plants during vegetation and after harvest. Thus, grey mould negatively impacts the quantity and quality of crop production, influencing all food chains including those of humans. *B. cinerea* causes losses of more than 1000 plant varieties, including 500 important crops [1]. Moreover, *Botrytis*, as a perfectly adapted pathogen, remembers all information about light because its biological clock monitor makes important decisions in the development of its host plant infection [2]. Its fungal colony and disease severity depend on the evolutionary trajectory and lifestyle of the fungus, as well as the predictability of the inputs in terms of host plant inches and the cost benefits for the fungus of predictable behaviour [3]. The susceptibility of plants to *B. cinerea* is mainly due to its attack strategy and nutritional habits that consist of direct invasion of a cell wall or lesions using fruiting bodies [4,5,6]. It seems like grey mould’s habits of invasion have improved its skills to survive during adverse environmental conditions. Penetration by *B. cinerea* harms plants, causing a grey mould infection with a specific smell, thriving in various plant tissues even if they are lifeless [7]. As with other diseases, grey mould’s infectious process has different stages. There are three main stages of the infectious process of *Botrytis cinerea*: early (0–36 h past inoculation), intermediate (36–48 h after inoculation), and late (>48 h after inoculation). Irreversible processes occur inside host plant cells during the early stage of grey mould infection [8].

One of the more than 500 susceptible crops for grey mould is green-leaf lettuce (*Lactuca sativa* L.). Leafy vegetables are often grown in controlled environments, such as greenhouses or growth chambers, so that fresh food can be enjoyed throughout the year, especially in areas of middle latitudes with seasonality [9,10]. Controlled environment agriculture (CEA) systems can produce fresh, nutrient-dense fruits and vegetables such as leafy greens consistently year-round in a climate-controlled environment (e.g., growth chambers) with significantly fewer resources [11]. Correspondingly, growth chambers, where moisture, temperature, and other nutritional levels are suitable for pathogenic fungi development and spreading, are the source of many challenges. Moreover, requirements for safe and healthy food production are quite high nowadays. In addition, horticulture is forced to limit fungicides because of fungal pathogens’ resistance and environmental pollution related to horticultural waste. *B. cinerea* has adapted to most chemical fungicides, inducing its resistance and causing negative impacts on the environment [7,12,13]. On the other hand, grey mould adaptation to pesticides contributes to an increasing interest in alternatives to chemical fungicides for bunch rot management [14]. A possible alternative is an intelligent usage of artificial lighting to protect plants with few or no chemicals.

Admittedly, light performs an overall vital role across the Earth. The main point is that light’s radiation affects living organisms differently. Generally, plants and some algae are photoautotrophic and convert light into chemical energy due to the production of organic compounds. Meanwhile, the same light may negatively impact pathogenic microorganisms such as the fungus *Botrytis cinerea*. In the case of host plant and fungus interaction, the consequences depend on the light’s intensity, spectrum, wavelengths, and photoperiod, as well as environmental conditions, host plant variety, pathogenic fungus species, and the time of the day. Consequently, the plant must receive enough light to produce passive and active response materials to resist and exist. Nonetheless, high-pressure sodium (HPS) lighting systems are a sufficiently common occurrence in horticulture. Unfortunately, HPS illumination is not an environmentally friendly supplementary lighting tool; moreover, it is quite expensive due to the increased electricity costs. Nowadays, evidence of LED usage’s benefits has been proven by various scientific studies because of the possibility of controlling its parameters such as wavelengths or radiation intensity and obtaining a higher quality of crop production with a reduction in energy costs [9,15,16,17]. For instance, leafy vegetables, such as Chinese broccoli and ice plants, showed the highest shoot productivity associated with a more significant leaf number and rapid leaf area development when 16% and 10% of blue LED, respectively, was supplemented with red LED [18]. Meanwhile, the causative agent of grey mould—*Botrytis cinerea*—reacts to blue light by forming sterile hyphae [2]. Also, red light disturbs the re-promotion of conidiation after blue light inhibition and decreases the amount of conidiation in far-red light-affected colonies [19]. Considering the research undertaken in further studies, LEDs impact plants and fungi.

As instruments of non-destructive methods, vegetation indices from different transformations of reflectance were developed to determine the contents of basic plant pigments [20]. Furthermore, vegetation indices are essential for studying plant health and useful for modern measuring techniques to evaluate it [21]. For example, the concentration of specific leaf pigments is directly proportional to the relative index. In the last decade, researchers have developed vegetation indices to detect disease stages since several symptoms have unique fluctuations in band reflection. Some symptoms affect the pigments or chlorophyll content, which then affect the green band’s reflection. Thus, reflection affect the bands in 500–600 nm [22]. Vegetation indices are functional for crop distinction in land cover applications for identifying canopy species in tropical forests, for detecting leaf and plant biophysical and biochemical properties, and for detecting plant stress and diseases [23]. For instance, regarding diseases, the Normalized Difference Vegetation Index (NDVI) was investigated as the most common vegetative index to determine infected plant tissue and used by most researchers to measure the density and health of plant vegetation in a specific area [24,25,26].

Naturally, using spectroscopic instruments is not without difficulties and challenges [27]. The precision and usage of optical imaging with sensors have specific needs regarding plant disease detection in horticulture [20]. Firstly, early disease detection needs to be accurate enough. Diseases need to be easily differentiated from other diseases and abiotic stress. Indeed, the influence of pathogenic fungi and the impact of abiotic stress can be sufficiently similar [28]. Therefore, it is necessary to study leaf reflectance bands and specific fungal activity behaviour before taking measures. Hence, the vegetation indices and plant phenotype should be tested and analysed many times in the long term. Secondly, the funds for practical usage should be available for producers. Usually, many investigations are performed in laboratories and incur considerable costs. The third but not the least important feature is applicability for rapid detection. Adaptability and convenience for users to obtain groups of specific plants may significantly affect spectral sensing methods for disease detection [29]. Doubtless, the speed of early disease detection using spectroscopic methods may influence successful and rapid diagnosis because of the fungal growth and development rate [30]. Many optical indices are suitable for detecting variations in the reflection of non-inoculated and inoculated plants. However, there is no disease-specific vegetation index. Hence, more vegetation indices must be monitored in detail [31]. The aim of this study was the potential of using vegetation indices for the early detection of grey mould and evaluating their changes during disease development in lettuces grown under different lighting conditions.

## 2. Results

**Figure 1:**
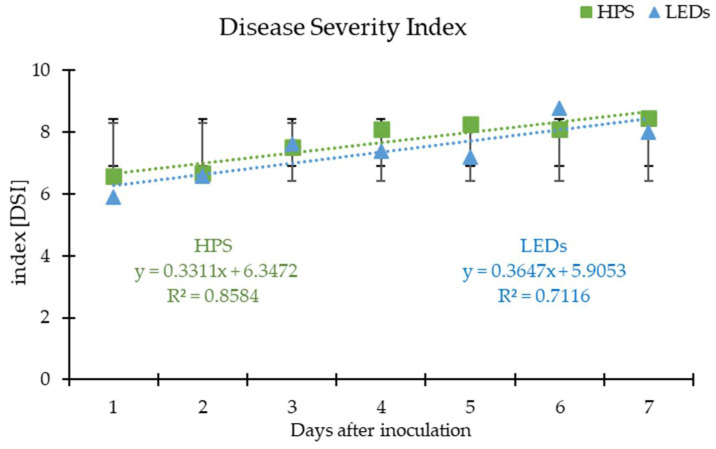
Disease severity index variation on lettuce in vivo grown under high-pressure sodium (HPS) and light-emitting diode (LED) lamps; coefficients for linear regression equations are presented in this figure, in the form of y = ax + b, and their correlation coefficients (R^2^).

The *B. cinerea* disease severity index (DSI) increased proportionally under HPS and LED irradiation (Figure 1). The correlation coefficients of HPS (0.85840) and LEDs (0.7116) were strong under both lighting systems, which indicated a strong association between lighting conditions and grey mould infection, but HPS was affected more significantly.

Lesions on leaves caused by *B. cinerea* appeared the first day after inoculation (DAI). The *B. cinerea* DSI under HPS reached 6.58 and that under LEDs reached 5.92 on the first day of DAI. However, on the second and the third DAI, DSI under HPS lamps were similar, 6.66 and 6.60, respectively. At the same time (second and third DAI), *B. cinerea* DSI on lettuce under LEDs was 6.6 and 7.63, respectively.

**Figure 2:**
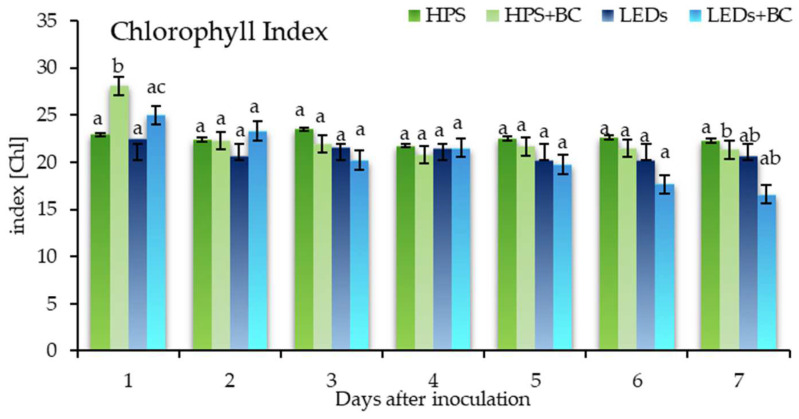
Chlorophyll index of green-leaf lettuce. Index of non-inoculated lettuce grown under high-pressure sodium lamps—HPS; lettuce inoculated with *B. cinerea* and grown under HPS—HPS + BC; non-inoculated lettuce grown under light-emitting diodes—LEDs; lettuce inoculated with BC and grown under LEDs—LEDs + BC: non-inoculated lettuce was selected as control; according to Duncan ‘s multiple range test, means with different letters are significantly different at the *p* < 0.05 level. Error bars show the standard deviation.

The chlorophyll index (Chl) of the inoculated lettuce was significantly higher than the non-inoculated lettuce grown under HPS lighting on the first DAI. Under the same environmental circumstances, except for other radiation (LEDs), the chlorophyll index of the inoculated lettuce was significantly lower than the non-inoculated lettuce under HPS irradiation on the first DAI (Figure 2). Thus, non-inoculated plants grown under both lighting conditions maintained the same trend but had significant differences from the inoculated lettuce.

Meanwhile, the chlorophyll index from the second to the sixth DAI did not show significant differences under different radiation conditions or between inoculated and non-inoculated lettuces. Although significant differences emerged on the seventh DAI under HPS radiation, the disease of grey mould had already progressed to a late stage.

The chlorophyll pigment PSSRb (Figure S1b) of inoculated lettuce (5.283) was significantly lower than non-inoculated (5.884) ones under HPS radiation. Lighting conditions did not reveal significant differences.

CRI2 (Figure S2a) was much more accurate in detecting grey mould disease on experimental green-leaf plants and significantly varied between inoculated and non-inoculated lettuce under both lighting conditions (HPS and LEDs). CRI2 data revealed that carotenoids decreased after infection, though pigments can increase because of experienced pathogenic fungus stress. SIPI (Figure S2b) of inoculated lettuce (0.686) was significantly lower than the same index of non-inoculated lettuce (0.704) under HPS radiation on the DAI. SIPI that measured inoculated and non-inoculated green-leaf lettuce grown under LEDs did not expose any significant differences. At the same time, vegetation index FRI (Figure S2c) maintained the same trend—flavanols of inoculated lettuce increased on the DAI with *Botrytis cinerea.* The vegetation index FRI indicated significant differences in DAI, but it goes beyond the grey mould’s early-stage limits.

**Figure 3:**
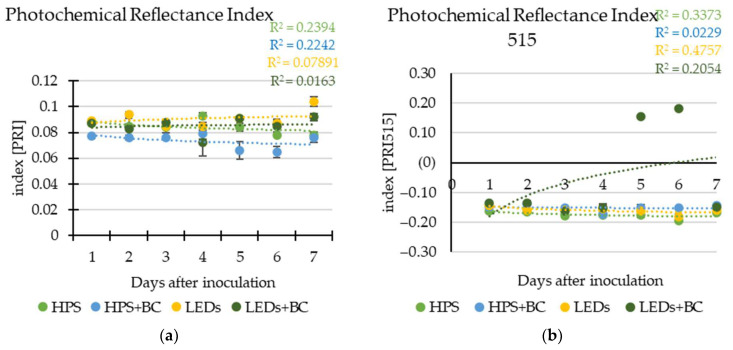
Xanthophyll indices of lettuce: (**a**) PRI, Photochemical Reflectance Index; (**b**) PRI515, Photochemical Reflectance Index 515. Index of non-inoculated lettuce grown under high-pressure sodium lamps—HPS; lettuce inoculated with *B. cinerea* and grown under HPS—HPS + BC; non-inoculated lettuce grown under light-emitting diodes—LEDs; lettuce inoculated with BC and grown under LEDs—LEDs + BC; non-inoculated lettuce was selected as control; error bars show the standard deviation. Coefficients for logarithmic regression equations are presented in this figure, in the form of y = a + b × ln(x), and their correlation coefficients (R^2^).

PRI (Figure 3a) showed the same trend as SIPI (Figure S2b)—significant differences between inoculated and non-inoculated lettuces revealed only on plants grown under HPS lighting. The vegetation xanthophyll or Photochemical Reflectance Index PRI515 (Figure 3b) indicated quite normal photosynthetic activity with interval values from −0.136 to −0.194 for the 7 days of the experiment. Generally, PRI515 values around 0 mean a normal amount of the photosynthesis pigments xanthophylls. For example, the PRI515 of inoculated (−0.149) lettuce was significantly higher than non-inoculated (−0.162) lettuce grown under HPS lighting at the early stage of grey mould infection.

Accordingly, colouration index B (Figure S3d) decreased after artificial grey mould infection under HPS lighting, whereas the B index of inoculated (1.101) experimental lettuce was significantly lower than non-inoculated (1.126) lettuce under HPS radiation on the DAI.

Meanwhile, the NDVI (Figure S4a) as greenness and disease indices of inoculated (0.751) experimental lettuce were significantly lower than the NDVI of non-inoculated (0.765) plants under HPS lamps. However, the same NDVI revealed significant reliability between the different lighting. The PSRI (Figure S4b), as a reliable plant health index, as well as the CRI2 (Figure S2a) indicated grey mould caused *B. cinerea.* Significant differences between inoculated and non-inoculated lettuces appeared under HPS and LEDs lamps. The HI (Figure S4c) is a reliable index for plant health monitoring. The HI of inoculated (0.054) lettuce significantly declined compared with non-inoculated (0.063) lettuce grown under HPS lamps. Also, the specific disease index for individual study (fD) (Figure S4d) of inoculated (0.658) lettuce was significantly lower than non-inoculated lettuce (0.667) grown under the HPS lighting system. Based on the data received, fD may reliably indicate the first signs of the disease, including grey mould on the first DAI involved. Looking at the early stage of grey mould, significant differences in WBI (Figure S4e) were exposed between non-inoculated (0.738) and inoculated (0.700) lettuces grown under HPS lighting. The WBI of inoculated lettuce was significantly lower than non-inoculated lettuce due to reduced leaf water content. The reliability between the different lighting conditions was also significant: the WBI of non-inoculated and inoculated lettuces grown under LEDs was significantly lower than those grown under HPS lamps after the DAI with *Botrytis cinerea*. Meanwhile, RVSI (Figure S4f), the common crop health index for monitoring and detecting vegetation stress index, varied from −1.8 to −2.0 for the entire duration of the experiment. As the grey mould progressed, RVSI values tended to decline. For instance, the RVSI of inoculated (−1.934) lettuce significantly declined compared to non-inoculated (−1.917) lettuce grown under HPS radiation on the DAI.

**Figure 4:**
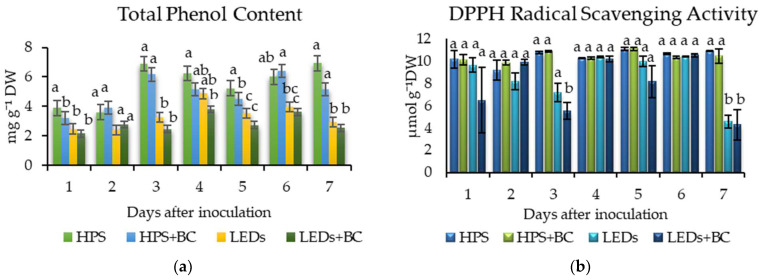
(**a**) The total phenolic content (TPC); (**b**) 2.2-diphenyl-1-picrylhydrazyl (DPPH) radical scavenging activity. TPC and DPPH of non-inoculated lettuce grown under high-pressure sodium lamps—HPS; lettuce inoculated with *B. cinerea* and grown under HPS—HPS + BC; non-inoculated lettuce grown under light-emitting diodes—LEDs; lettuce inoculated with BC and grown under LEDs—LEDs + BC; non-inoculated lettuce was selected as control; DW means ‘dry weight’; the data were processed using analysis of variance (ANOVA). Duncan’s multiple range test means with other letters are significantly different at the *p* < 0.05 level. Error bars show the standard deviation.

The destructive biochemical analysis of total phenolic content (TPC) and 2.2-diphenyl-1-picrylhydrazyl (DPPH) free radical scavenging activity was conducted to verify and compare whether the destructive disease detection method corresponds to the non-destructive one (Figure 4). In contrast to our earlier findings with the chlorophyll index (Figure 2), the TPC of lettuce grown under HPS and LEDs decreased on the first DAI under HPS and LED lighting. The TPC of inoculated and non-inoculated lettuces grown under HPS lamps was significantly higher than lettuces grown under LEDs on the third DAI. The early grey mould detection data on the first DAI outlined that the TPC of inoculated lettuce under illumination with HPS lamps was significantly reduced after the first DAI. According to the results, the destructive TPC method only indicated significant differences between different radiation conditions, except on the first DAI.

According to the biochemical TPC (Figure 4a) analysis circumstances of the 7-day experiment, inoculated lettuce grown under LED irradiation produced less TPC than lettuce grown under HPS. The TPC of inoculated (3.23 mg g^−1^) lettuce was significantly lower than non-inoculated (3.9 mg g^−1^) lettuce under HPS after the first DAI. The third DAI showed significantly decreased TPC of lettuce (inoculated (2.46 mg g^−1^) and non-inoculated (3.28 mg g^−1^)) under LEDs than experimental lettuce grown under HPS (inoculated (6.16 mg g^−1^) and non-inoculated (6.89 mg g^−1^)). The same trend remained for the next 4 days until the end of the experiment. The DPPH (Figure 4b) radical scavenging activity of inoculated (5.5 mg g^−1^) lettuce grown under LEDs revealed a significant difference on the third DAI. Meanwhile, the DPPH of inoculated (10.88 mg g^−1^) and non-inoculated (10.75 mg g^−1^) lettuces grown under HPS was significantly higher than lettuces grown under LEDs on the third DAI. The same DPPH tendency was approximately replicated on the seventh DAI.

## 3. Materials and Methods

### 3.1. Lettuce Growth Conditions

The experiments were performed in controlled environment chambers at the Institute of Horticulture, Lithuanian Research Centre for Agriculture and Forestry.

Green-leaf lettuces (*Lactuca sativa* L. cv. Little Gem) were obtained from Klaus Laitenberger/Green Vegetable Seeds, Alderwood, Eden. Seeds were sown in plastic pots (9 × 9 cm), and three seeds were placed in one plastic vessel containing peat substratum PROFI-1 (Durpeta, Šepeta, Lithuania). The pH (H_2_O) of turf substratum was 5.5–6.5, and the medium amount of macro- and micro-elements (mg L^−1^) were as follows: N, 110; P_2_O_5_, 50; K_2_O, 160; Ca, 242; Mg 29.5; S, 212; Fe, 1.7; Mn, 0.5; Cu, 31; B, 2; Zn, 1.6. Lettuce plants were fertilised with Nutrifol (brown NPK, where nitrogen (N)—14, phosphorus (P)—9, and potassium (K)—25, and green NPK, where N—8, P—11, and K—35) (YARA Poland, Sp. z.o.o., Szczecin, Poland), calcium nitrate (Yara Suomy Oy, Helsinki, Finland), magnesium sulphate (Zlotniki, S.A., Wrocław, Poland), and ammonium nitrate (PULAWY, S.A., Puławy, Poland), concerning the growth stage at the third week. The water was acidified with nitric acid. The final salt concentration was EC 2.8–3.0, acidity—pH 5.5–5.8. Day and night temperatures were set at 21 ± 2/17 ± 2 °C. Experimental lettuces were grown under high-pressure sodium (HPS; SON-T Agro, 400 W, Philips, Eindhoven, The Netherland) lamps before the experiment until BBCH21. Also, plants selected for the LED illuminating chamber were kept for 2–3 days under LED lamps (Heliospectra RX30, Gothenburg, Sweden) to adapt plants and watered as needed.

### 3.2. Light Treatments

The experiment was conducted under high-pressure sodium (HPS; SON-T Agro, 400 W, Philips, Eindhoven, The Netherland) irradiation and light-emitting diode (LED; Heliospectra RX30, Gothenburg, Sweden) lamps (Table S1). HPS and LED lighting were generated from 6 a.m. until 10 p.m. (16 h photoperiod, 8 h at dark regime) in different growth chambers. The light intensity of the HPS and LEDs was expressed by photon flux density (PPFD) and was set at 200 m^−2^ s^−1^. PPFD was measured using a photometer RF-100 with head G. PAR-100 (Sonopan, Bialystok, Poland). Configuration of the LEDs’ irradiance was measured using a FLAME-S-UV-VIS-ES spectrometer (Ocean Optics, Ostfildern, Germany).

### 3.3. Botrytis cinerea Preparation

*Botrytis cinerea* (Pers.) Fr. (grey mould) isolate (LT13B_FRA_76) was obtained from the Lithuanian Research Centre for Agriculture and Forestry, Institute of Horticulture, Laboratory of Plant Protection. The fungus before experiments was stored in a refrigerator at +4 °C, then was stored in a refrigerator at +4 °C. *B. cinerea* was maintained on potato dextrose agar (PDA; Liofilchem, Roseto degli Abruzzi, Italy) at 22 ± 2 °C in a thermostat. For lettuce inoculation, we used 7-day-old, 5 mm diameter *B. cinerea* taken from the periphery of the petri dish [7]. The central part of the lettuce leaf was inoculated.

### 3.4. Botrytis cinerea Inoculation on Lettuce In Vivo

The experimental non-inoculated and inoculated lettuces were separated by a two-sided agro-sheet in the growth chamber with an HPS lighting system at the beginning of the fourth week from sowing at BBCH 21. The non-inoculated and inoculated lettuces were separated in the second chamber under LEDs. Each leaf was inoculated with 5 mm diameter *B. cinerea* mycelium plug side-down on the upper leaf vein. Each leaf vein was scratched with a sterile wooden stick before inoculation. The *B. cinerea* plug was inoculated on three leaves of each plant. Inoculated and non-inoculated lettuces were maintained at 70 ± 10% relative humidity (RH) during the experiment. The disease that spread on the lettuces under different lighting treatments (HPS, high-pressure sodium lamps; LEDs, light-emitting diodes) was analysed daily from 1 to 7 days after inoculation (DAI). Non-inoculated lettuces grown under the HPS, and LEDs were selected as controls (non-inoculated). The diseased inoculated leaf area on each leaf was estimated visually. A score was assigned to each leaf according to the percentage of the surface leaf area covered with lesions on a scale of 1–12, where 1 = 0%, 2 = >0–<3%, 3 = >3–<6%, 4 = >6–<12%, 5 = >12–<25%, 6 = >25–<50%, 7 = >50–<75%, 8 = >75–<87%, 9 = >87–<94%, 10 = >94–<97%, 11 = >97–<100%, and 12 = lettuce leaf completely damaged. [32]. The *Botrytis cinerea* disease severity index (DSI) was calculated according to the formula [33]:

$$\mathrm{DSI}=\sum^{\;}\frac{\left(n\times \left(\mathrm{level}\;\mathrm{of}\;B.\;cinerea\;\mathrm{disease}\;\mathrm{severity}\right)\right)}{\mathrm{Total}\;\mathrm{inoculated}\;\mathrm{leaves}\;\mathrm{per}\;\mathrm{DAI}},$$

where ‘n’ means a group of lettuce leaves with a specific level of grey mould which caused the damage; DSI was estimated for each 7-day experiment.

### 3.5. Non-Destructive Measurements

The leaf chlorophyll index was measured by a chlorophyll/flavonol meter (Dualex^®^4, Scientific, Force-A, France) varied under different light conditions. An optical spectrophotometer (CID Bio-Science, Camas, WA, USA) [34] was used to measure spectral vegetative indices. Meanwhile, the following spectral vegetation indices in lettuce leaves were determined in vivo using a CI-710s Spectra Vue Leaf Spectrometer (CID Bio-Science, USA) from 9 a.m. to 12 a.m. with compatible software: Anthocyanin Reflectance 2 (ARI2); Carotenoid Reflectance (CRI2); Structure Intensive Pigment (SIPI); Flavonol Reflectance (FRI); Greenness (G); Greenness G2 (G2); Redness (R); Blue (B); Blue Green Index (BGI2); Browning Reflectance (BRI2); Lichtenthaler Index (LIC1); Pigment Specific Simple Ratio (PSSR-a and PSSR-b); Gitelson and Merzlyak (GM1 and GM2); Zarco-Tejada–Miller Index (ZMI); Normalized Difference Vegetation (NDVI); Simple Ratio (SR); Red-Eye Vegetation Stress Index (RVSI); Photochemical Reflectance Indices (PRI and PRI515); Water Band (WBI); specific disease index for individual study (fD), Healthy (HI); Plant Senescence Reflectance (PSRI); Vogelmann Red Edge Index (VREI1); Red Edge Normalized Difference Vegetation (RENDVI); and Modified Red Edge Simple Ratio (MRESRI). The leaf spectrometer was calibrated before measurements. Then, the dry leaf was placed inside the leaf clip. Spectrophotometric measurements were performed every day of the experiment on five living leaves of each lettuce, with three single measurements per leaf. Reflection spectra from the leaves were used to calculate vegetation indices according to producers’ equations (Table S2) [24,35,36,37,38,39,40,41,42,43,44,45,46,47,48,49,50,51,52,53,54,55].

### 3.6. Determination of Total Phenolic Content

The total phenolic contents were determined from various fresh plant tissues and 80% ice-cold methanol (Sigma-Aldrich, St. Louis, MO, USA) extracts. A total of 0.5 g of frozen lettuce tissue was homogenised in a ceramic mortar with 5 mL of methanol solution and transferred to a 15 mL polypropylene conical centrifuge tube (Labbox Labware S.L., Barcelona, Spain). The prepared total phenolic supernatant was identified spectrophotometrically with slight modifications. The extract was incubated at 4 °C for 1 day. At that time, samples were centrifuged (Hermle Z 300 K, Hermle Labortechnik, Wehingen, Germany) at a relative centrifugal force of 4000 rpm min^−1^ for 10 min at room temperature. The extract was filtered through a 70 mm qualitative filter paper (Frisenette ApS, Knebel, Denmark). Firstly, 100 µL of the filtrate was diluted with 200 µL of 10% (*v*/*v*) Folin & Ciocalteu‘s reagent and mixed thoroughly. Secondly, 800 µL of 700 mM 7.5% Na_2_CO_3_ dissolved in distilled water was added. The absorbance of the samples was measured at 765 nm against distilled water as a blank using a spectrophotometer CamSpec M501 (Spectronic CamSpec Ltd., Garforth, UK) after 20 min. The total phenolic content was calculated using gallic acid as a standard. Meanwhile, the total phenolic contents in those supernatants were expressed as equivalent gallic acid (mg g^−1^ extract) based on the regression equation of the calibration curve (R^2^ > 0.95). The TPC analysis was performed 7 DAI.

### 3.7. Evaluation of DPPH Free-Radical Scavenging Activity

The radical scavenging activity of 2.2-diphenyl-1-picrylhydrazyl (DPPH) was evaluated spectrophotometrically. A total of 100 µL of 80% methanol extracts used for the TPC assay was diluted with 1 mL of 60 µM DPPH solution. Absorbance was measured after 16 min using the spectrophotometer (M501, Spectronic Camspec Ltd., Leeds, Yorkshire, UK) at 515 nm. Data are presented as the mean of three analytical samples to scavenge DPPH free radicals (in µmol g^−1^) on a dry basis of the plant. The DPPH free-radical scavenging activity analysis was performed 7 days after inoculation (DAI).

### 3.8. Statistical Analysis

Statistical analysis was performed using XLSTAT 2022.3.1 (Addinsoft, Paris, France) software packages. The Duncan method was used for significant tests and ANOVA via the multiple comparison method (*p* ≤ 0.05). Figures were constructed using Microsoft Excel for Microsoft 365 MSO (ver. 2301 Build 16.0.16026.20196).

## 4. Discussion

As a polyphagous and necrotrophic microorganism, grey mould, caused by *B. cinerea*, also needs light and oxygen to exist because of its demand for the autotrophic organisms of plants suitable for its nutrition. Considering early diagnostic methodology, grey mould detection includes only the first and second DAI [8]. Naturally, *B. cinerea* is adapted to solar light, but some components of the photoperiod and photosynthetic photon flux density may impact its growth and development. Correspondingly, irradiation with 405 nm light significantly reduced lesion diameter 4 days after inoculation compared with control [56]. Therefore, investigations from the 1970s revealed that the fungus responds to different light wavelengths, spanning those from near-UV to far-red light [57]. Results showed that the far-red radiation-induced susceptibility in tomato plants is not specific to *B. cinerea*, as supplemental far-red increased symptom development caused by an array of pathogens [58]. Moreover, not all wavelengths of light are used to suppress grey mould formation, and the ability to spread reflects the primary symptoms of the disease. Currently, in this study, green-leaf lettuce inoculated with *B. cinerea* plugs were significantly different depending on total HPS (200 µmol m^−2^ s^−1^) and LED lighting (purple, with 2 µmol m^−2^ s^−1^; blue, with 40 µmol m^−2^ s^−1^; green, with 14 µmol m^−2^ s^−1^; orange, with 24 µmol m^−2^ s^−1^; red, with 108 µmol m^−2^ s^−1^; far-red, with 12 µmol m^−2^ s^−1^).

As in our study with lettuce, the contents of chlorophyll and carotenoids were induced in the leaves of cucumber seedlings under different LED light models in another study [59]. The increase in pigments possibly changed because of the photosynthetic area and water band reduction during infection. This study confirmed that the WBI (Figure S4e) tends to decrease due to grey mould infection. The WBI of non-inoculated (0.0738) lettuce was significantly higher than inoculated (0.0700) lettuce under HPS lighting on the first DAI. Not long afterward, the amount of pathogen inhibitors (e.g., flavanols and anthocyanins) increased mainly on the first to third DAI. Our experiment confirms the mentioned results. Therefore, the FRI of inoculated lettuce increased dramatically on the second DAI under HPS and LEDs but did not reveal significant differences between lighting conditions. The ARI2 of inoculated lettuce maintained the same trend as the second DAI, except under HPS radiation, from which it was significantly distinguished. The increase in the Flavanol Reflectance Index (FRI) means a reduced efficiency of photosynthesis, which manifests in the lack of water [60]. Therefore, FRI is inversely proportional to WBI. Correspondingly, the knowledge that lettuce lost its water content and significantly increased the flavanol amount makes it possible to assume that the plant was stressed and pushed to activate its defence system. Respectively, our study confirms that the CRI2, NDVI, PRI, and WBI vegetation indices were significantly distinguished after *B. cinerea* inoculation on green-leaf lettuce (*Lactuca sativa* L.) at an early infectious stage only under HPS lighting. As it is known, pigments such as chlorophyll, carotenoid, and xanthophyll closely depend on light and photosynthetic area. The consequence of all this is a decrease in vegetation pigments. Therefore, pigments and their indices decrease, or an increase may reliably indicate the host plant’s condition. Our findings seem to show that lettuces likely became stressed against *B. cinerea*, increasing chlorophyll the first day after artificial infection under HPS lighting (Figure 2). A significant increase in the chlorophyll index for inoculated lettuce demonstrated the evidence of the previous statement. By contrast, the highest chlorophyll index of cucumbers were cultivated under red:blue (ratio 70:30) LEDs and light intensity 150 ± 2 µmol m^−2^ s^−1^, photoperiod 12 h [59]. Also, flavanols are naturally found as soluble phenolics, which act as a plant defence apparatus, and closely depend on light and pathogen invasion. In this paper, HPS radiation efficiently induced the process of flavanol formation that is expressed in the SIPI, FRI, and ARI2 indices (Figure S2). Inoculated lettuce (*Lactuca sativa* L.) leaves accumulated more flavanols under both lighting systems due to oxidative stress caused by *B. cinerea,* but the increase was insignificant. Hence, light’s manipulation allows for the control of plants’ qualitative properties [61].

In terms of plant indices, Vaštakaitė et al., 2021 found that the CRI, NDVI, and PRI of inoculated lettuce (*L. sativa* L. cv. Little Gem) significantly increased 18 h past artificial infection with *Botrytis cinerea* disk. Accordingly, the CRI of inoculated lettuce increased by 36%. In addition, the NDVI and PRI significantly increased (by 8 and 33%, respectively) in the same infected lettuce compared to non-infected lettuce 18 h after artificial infection [62]. Hence, the content of carotenoids, chlorophylls, and xanthophylls tended to increase after being stressed by *B. cinerea*. This supports that the CRI2, NDVI, PRI, and WBI vegetation indices were significantly distinguished after *B. cinerea* invasion on green-leaf lettuce (*Lactuca sativa* L.) at an early infectious stage only under HPS lighting. Also, the plant healthy index (HI) was sufficiently accurate to indicate grey mould, which shows significant differences between inoculated (0.054) and non-inoculated (0.063) lettuces (Figure S4c). Higher values of the HI indicate higher chlorophyll content, while lower values correspond to lower chlorophyll content. The HI was applicable in detecting all symptoms of necrotic lesions on the sugar beet leaf with an accuracy of 95.91%, and characteristic features were revealed as it was inoculated [24]. Other coloration indices such as B (Blue Index), BG1 (Blue-Green Index), and BR1 (Blue-Red ratios) were identified as the best detectors for *Verticillium* wilt (*Verticillium dahliae* Kleb) at the early stage, while the structural, modified simple ratio, plant disease indices NDVI and PRI515, and the chlorophyll and carotenoid indices appeared to be adequate detectors for the presence of moderate to severe damage. Chlorophyll indices such as GM1, GM2, PSSRa, and PSSRb showed significantly (*p* < 0.05) lower values on moderately and severely affected olive trees by *Verticillium* wilt compared with values estimated on asymptomatic trees [36].

Our results indicated that the CRI2, PRI515, and HI of inoculated lettuce with *Botrytis cinerea* were significantly lower under HPS lighting. The same indices CRI2, PRI515, and HI of inoculated and non-inoculated plants under LEDs had no significant differences. The PSSRb of inoculated lettuce was significantly lower than the control under HPS irradiation. Other physiological indices such as GM1 and GM2 detected grey mould infection on the third to seventh DAI and went beyond the early stage of the disease.

## 5. Conclusions

In this study, plant health indices such as NDVI, PSRI, HI, WBI, and RVSI, xanthophyll indices PRI and PRI515, carotenoid indices CRI2 and SIPI, chlorophyll index PSSRb, and coloration index B were able to detect *Botrytis cinerea* infection on green-leaf lettuce (*Lactuca sativa* L. cv Little Gem) at the first day after inoculation. However, fD and PSRI were identified as the best *B. cinerea* detectors under both lighting conditions (HPS and LEDs). According to our results, vegetation indices revealed more significant differences in inoculated lettuce grown under HPS lamps than non-inoculated ones. Therefore, the performance of LED components in this study did not reveal a significant positive influence on fungal disease suppression but induced the antioxidative properties of lettuce such as flavanols, carotenoids, and anthocyanins. Moreover, LEDs are more hopeful because of their environmentally friendly components. With factual evidence of supplemental lighting’s impact on disease control, further research on various LED compositions is needed.

## Data Availability

The data presented in this study are available in article.

## Supplementary Materials

The following supporting information can be downloaded at https://www.mdpi.com/2223-7747/12/23/4042/s1, Figure S1: chlorophyll (a,b) indices of lettuce; Figure S2: carotenoid, flavanol, and anthocyanin indices of lettuce; Figure S3: coloration indices of lettuce; Figure S4: disease indices of lettuce; Table S1: configuration of LEDs according to HPS; Table S2: vegetation indices used in this study and their formulas.
