# Supplementary material for: Vegetation Indices for Early Grey Mould Detection in Lettuce Grown under Different Lighting Conditions
Source: Plants (Basel). 2023 Nov 30;12(23):4042. doi: 10.3390/plants12234042 (PMC10871106; doi:10.3390/plants12234042)
Supplement: Supplementary file 1 [file plants-12-04042-s001.zip › plants-2712078-supplementary.pdf]

**Figure S1.** Chlorophyll (a+b) indices of lettuce. **(a)** PSSRa; Pigments Specific Simple ratio a; **(b)** PSSRb; Pigments Specific Simple Ratio b; **(c)** GM; Gitelson and Merzlyak Index; **(d)** GM2; Gitelson and Merzlyak 2; **(e)** ZMI; Zarco Tejada Miller (Red Edge) Index; **(f)** LIC1; Lichtenthaler's Index 1; **(g)** VREI; Volgelmann Red Edge Index; **(h)** SR; Simple Ratio Index. Index of non-inoculated lettuce and grown under high-pressure sodium lamps – HPS; Lettuce inoculated with *B. cinerea* and grown under HPS – HPS+BC; Non-inoculated lettuce grown under light-emitting diodes – LEDs; Lettuce inoculated with BC and grown under LEDs – LEDs+BC; Non-inoculated lettuce was selected as control; Error bars show standard deviation. Coefficients for logarithmic regression equations are presented in this figure, in the form of y = a + b×ln (x), and their correlation coefficients ($R^2$).

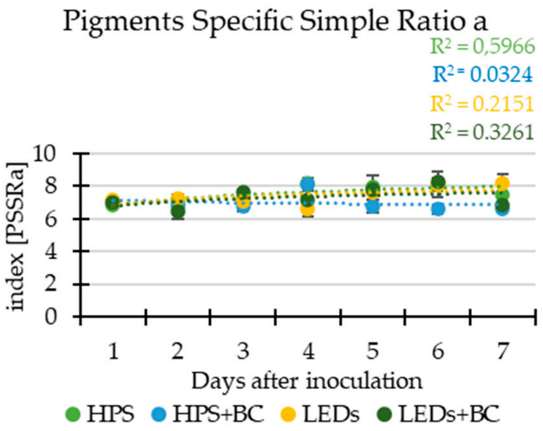


**(a)**

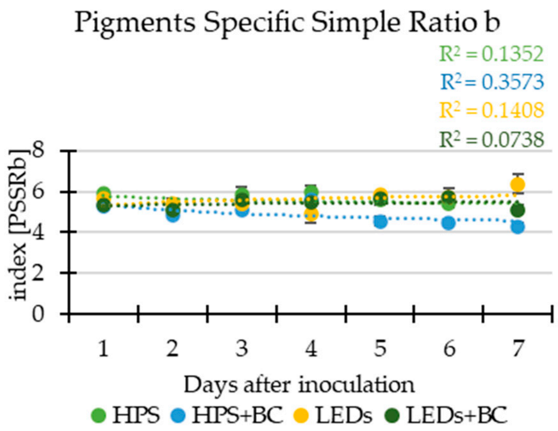


**(b)**

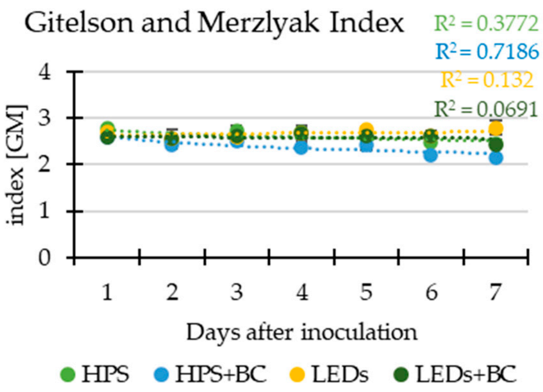


**(c)**

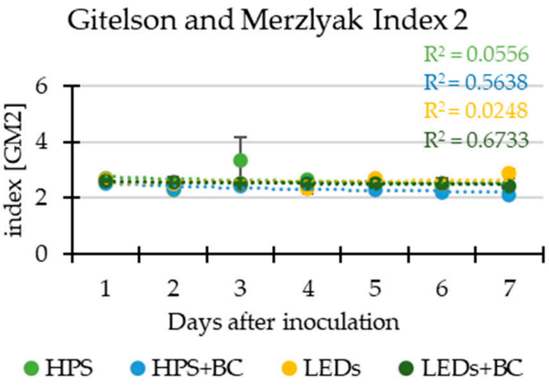


**(d)**

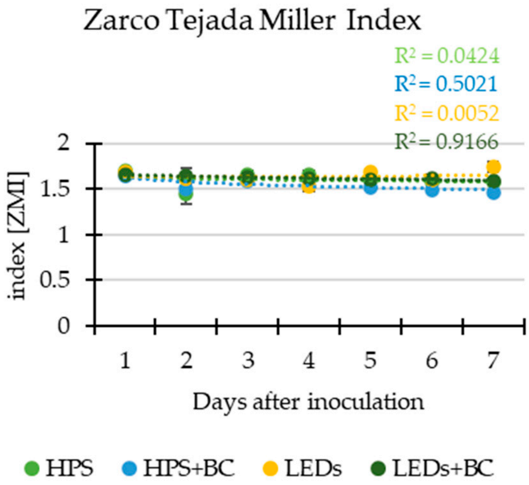


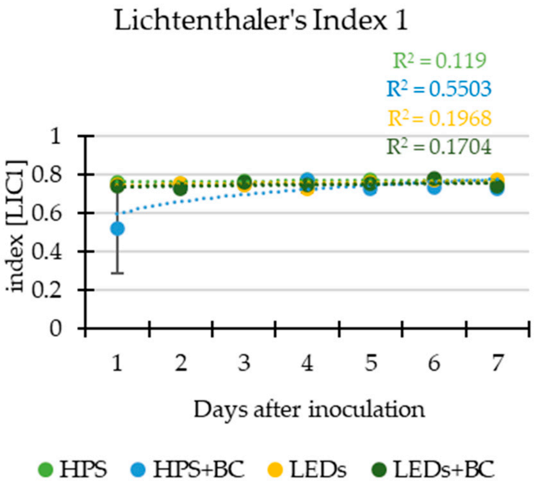

**(e)** **(f)**

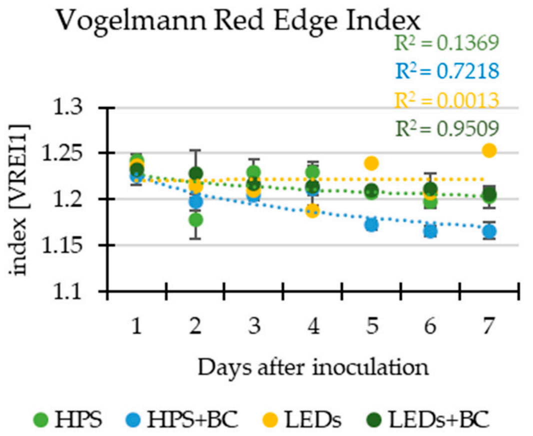


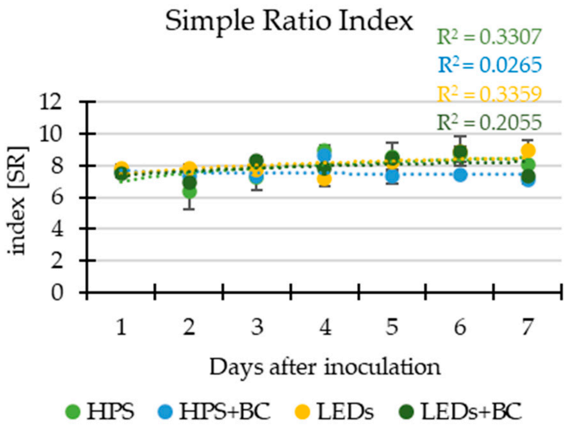


**(g)** **(h)**

**Figure S2.** Carotenoid, flavanol and anthocyanin indices of lettuce. **(a)** CRI2; Carotenoid Reflectance Index 2; **(b)** SIPI; Structure Intensive Pigment Index; **(c)** FRI; Flavanols Reflectance Index; **(d)** ARI2; Anthocyanin Reflectance Index 2. Index of non-inoculated lettuce and grown under high-pressure sodium lamps – HPS; Lettuce inoculated with *B. cinerea* and grown under HPS – HPS+BC; Non-inoculated lettuce grown under light-emitting diodes – LEDs; Lettuce inoculated with BC and grown under LEDs – LEDs+BC; Non-inoculated lettuce was selected as control; Error bars show standard deviation. Coefficients for logarithmic regression equations are presented in this figure, in the form of y = a + b×ln (x), and their correlation coefficients ($R^2$).

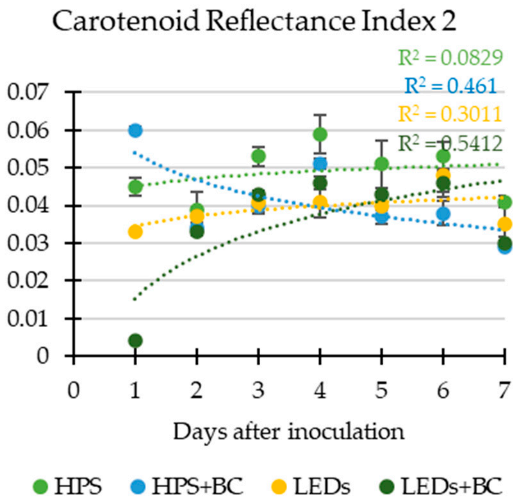


**(a)**

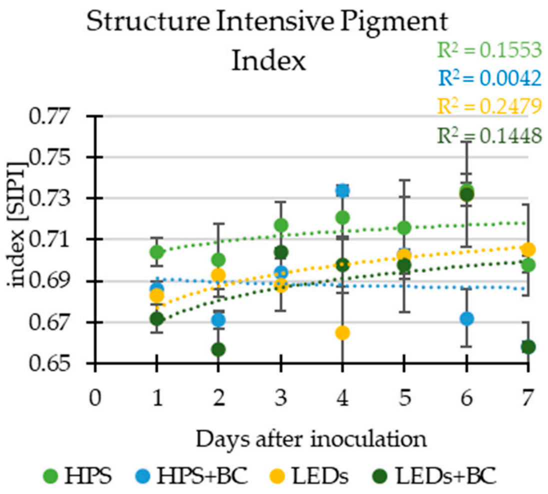


**(b)**

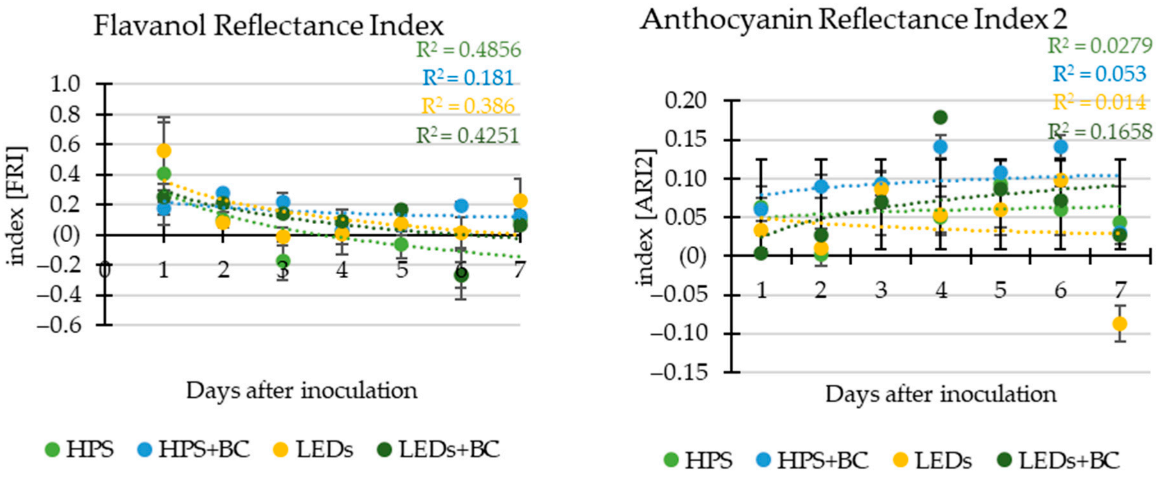


**(c)** **(d)**

**Figure S3**. Coloration indices of lettuce. **(a)** G; Greenness Index; **(b)** G2; greenness 2; **(c)** R; Redness Index; **(d)** B; Blue Index; **(e)** BGI2; Blue Green Pigment Index; **(f)** BRI2; Browning Reflectance Index. Index of non-inoculated lettuce and grown under high-pressure sodium lamps – HPS; Lettuce inoculated with *B. cinerea* and grown under HPS – HPS+BC; Non-inoculated lettuce grown under light emitting diodes – LEDs; Lettuce inoculated with BC and grown under LEDs – LEDs+BC; Non-inoculated lettuces was selected as a control; Error bars show standard deviation. Coefficients for logarithmic regression equations are presented in this figure, in the form of y = a + b×ln (x), and their correlation coefficients ($R^2$).

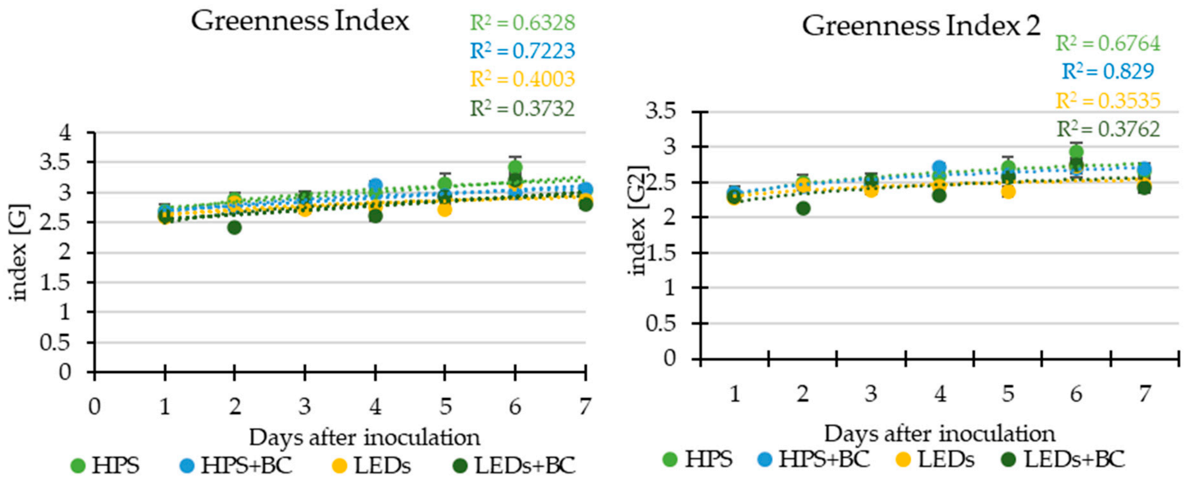


**(a)** **(b)**

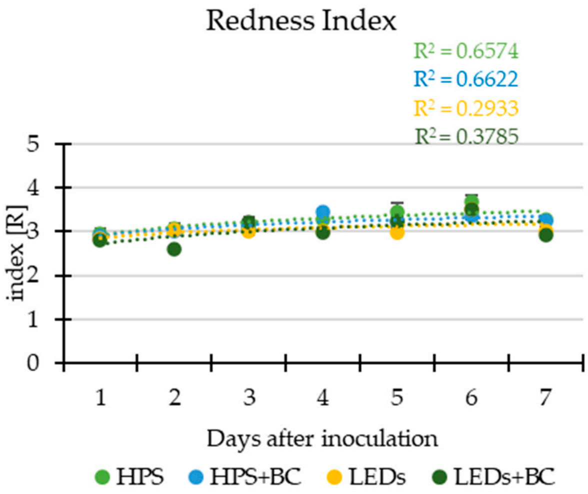


**(c)**

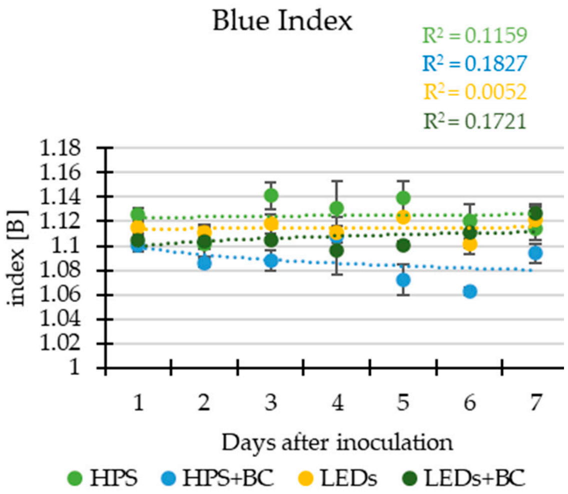


**(d)**

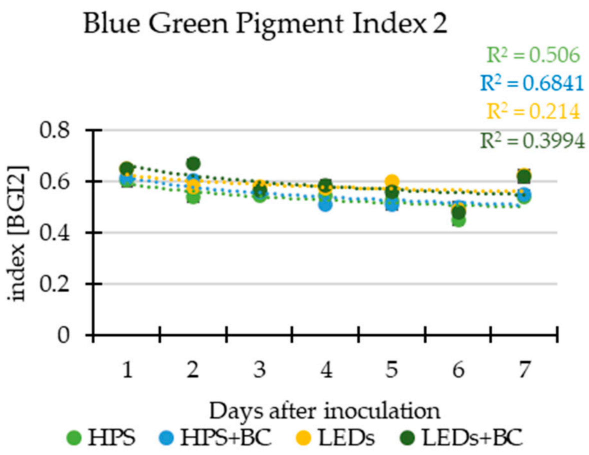


**(e)**

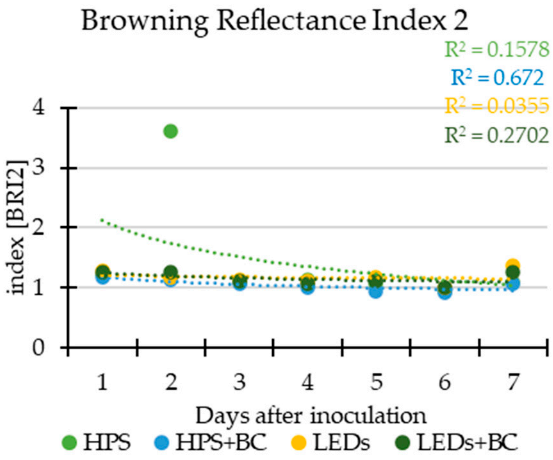


**(f)**

**Figure S4.** Disease indices of lettuce. **(a)** NDVI; Normalized Difference Vegetation Index; **(b)** PSRI; Plant Senescence Reflectance Index; **(c)** HI; Healthy Index; **(d)** fD; Specific disease index for individual study; **(e)** WBI; Water Band Index; **(f)** RVISI; Red-eye Vegetation Stress Index; **(g)** MRESRI; Modified Red Edge Simple Ratio Index; **(h)** RENDVI; Red Edge Normalized Difference Vegetation Index. Index of non-inoculated lettuce and grown under high-pressure sodium lamps – HPS; Lettuce inoculated with *B. cinerea* and grown under HPS – HPS+BC; Non-inoculated lettuce grown under light-emitting diodes – LEDs; Lettuce inoculated with BC and grown under LEDs – LEDs+BC; Non-inoculated lettuce was selected as control. Error bars show standard deviation. Coefficients for logarithmic regression equations are presented in this figure, in the form of y = a + b×ln (x), and their correlation coefficients ($R^2$).

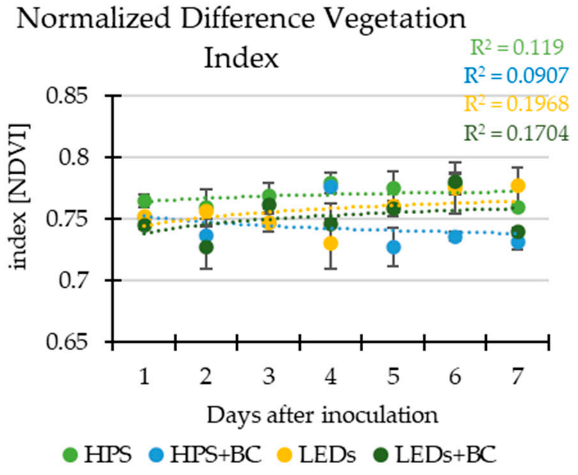
Normalized Difference Vegetation Index
R² = 0.119
R² = 0.0907
R² = 0.1968
R² = 0.1704
index [NDVI]
0.85
0.8
0.75
0.7
0.65
1 2 3 4 5 6 7
Days after inoculation
HPS HPS+BC LEDs LEDs+BC

**(a)**

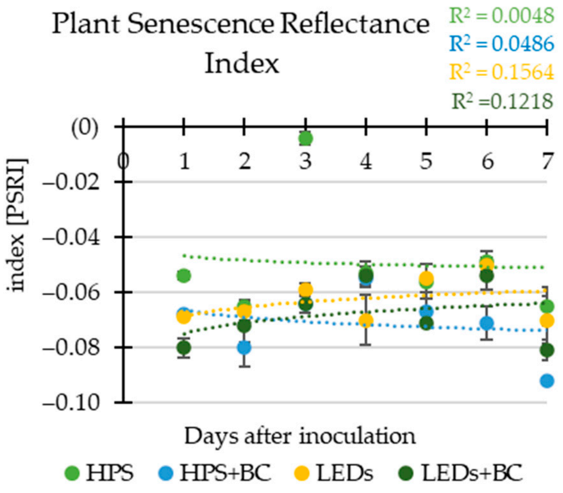
Plant Senescence Reflectance Index
R² = 0.0048
R² = 0.0486
R² = 0.1564
R² = 0.1218
index [PSRI]
(0)
−0.02
−0.04
−0.06
−0.08
−0.10
0 1 2 3 4 5 6 7
Days after inoculation
HPS HPS+BC LEDs LEDs+BC

**(b)**

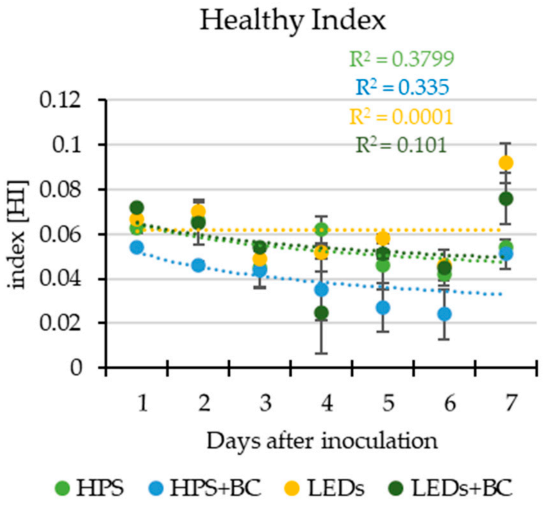
Healthy Index
R² = 0.3799
R² = 0.335
R² = 0.0001
R² = 0.101
index [HI]
0.12
0.1
0.08
0.06
0.04
0.02
0
1 2 3 4 5 6 7
Days after inoculation
HPS HPS+BC LEDs LEDs+BC

**(c)**

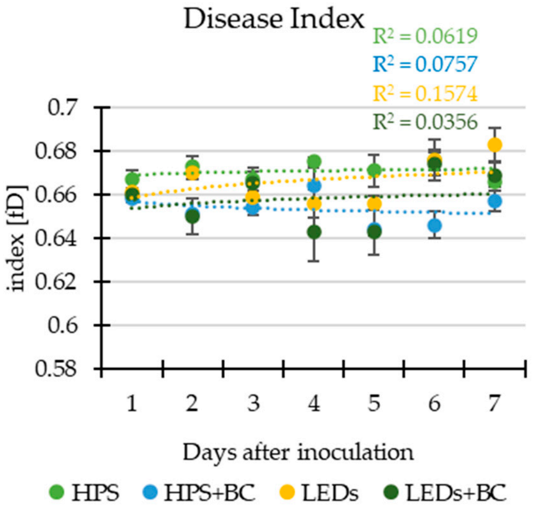
Disease Index
R² = 0.0619
R² = 0.0757
R² = 0.1574
R² = 0.0356
index [fD]
0.7
0.68
0.66
0.64
0.62
0.6
0.58
1 2 3 4 5 6 7
Days after inoculation
HPS HPS+BC LEDs LEDs+BC

**(d)**

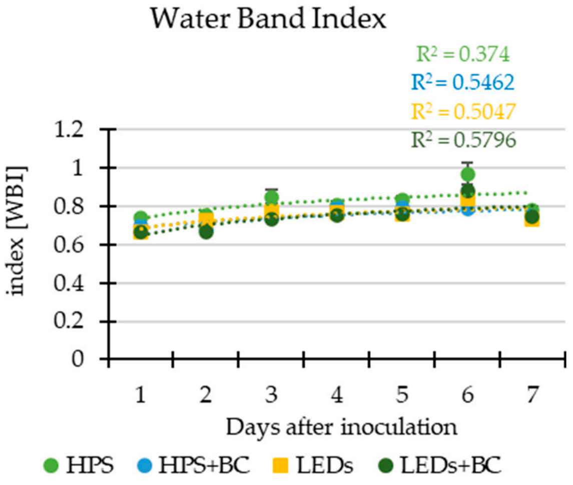
Water Band Index
R² = 0.374
R² = 0.5462
R² = 0.5047
R² = 0.5796
index [WBI]
1.2
1
0.8
0.6
0.4
0.2
0
1 2 3 4 5 6 7
Days after inoculation
HPS HPS+BC LEDs LEDs+BC

**(e)**

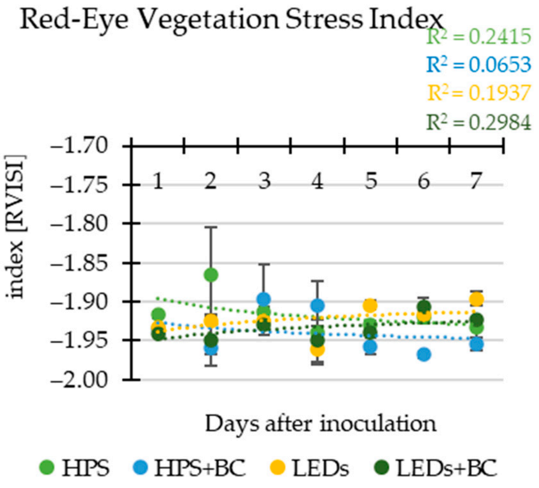
Red-Eye Vegetation Stress Index
R² = 0.2415
R² = 0.0653
R² = 0.1937
R² = 0.2984
index [RVISI]
−1.70
−1.75
−1.80
−1.85
−1.90
−1.95
−2.00
1 2 3 4 5 6 7
Days after inoculation
HPS HPS+BC LEDs LEDs+BC

**(f)**

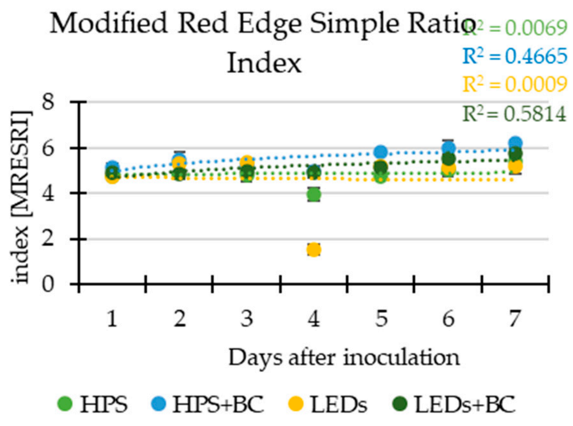


(g)

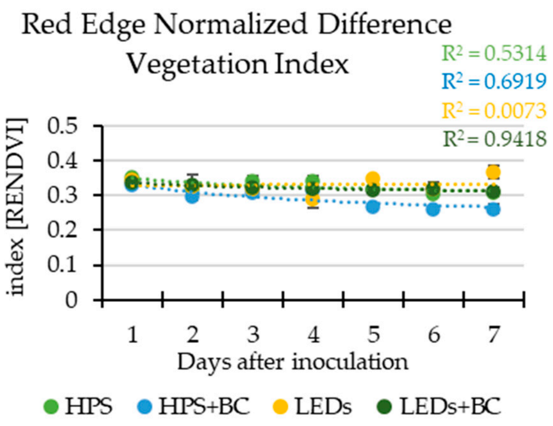


(h)

**Table S1**. Configuration of LEDs according to HPS.

| | **HPS spectrum** | Targeted spectrum | | **LEDs spectrum** | | Determined spectrum | |
|---|---|---|---|---|---|---|---|
| | | | | Possibilities | | | |
| Nanometers (nm) | From the general spectrum, % | From the general spectrum, % | µmol $m^{-2}$ $s^{-}$, while general PPFD 200 | LEDs wave-length | LEDs max stream | From the general spectrum, % | µmol $m^{-2}$ $s^{-}$, while general PPFD 200 |
| 360-760 | 100 | 100 | 200 | | | 100 | 200 |
| 700-760 | 6 | 6 | 12 | 735 (far-red) | 75 | 6 | 12 |
| 625-700 | 25 | 25 | 50 | | | | |
| 670-700 | 9 | | | | | | |
| 650-670 | 4 | | | 660 (red) | 125 | 54 | 108 |
| 625-650 | 13 | | | | | | |
| 590-625 | 29 | 29 | 58 | 620 (orange) | 24 | 12 | 24 |
| 550-590 | 24 | 11 | 22 | | | 0 | 0 |
| 490-550 | 7 | 7 | 14 | 530 (green) | 80 | 7 | 14 |
| 425-490 | 7 | 20 | 40 | | | | |
| 460-490 | 3 | | 0 | 450 (blue) | 100 | 20 | 40 |
| 425-460 | 4 | | 0 | 420 (violet) | 60 | | |
| 400-425 | 2 | 2 | 4 | 400 (purple) | 25 | 1 | 2 |
| 360-400 | 1 | 1 | n | 380 (purple) | 1.2 | | |

**Table S2**. Vegetation indices used in this study and their formulas.

| **Vegetation indices** | **Equation** | **Common and green** | **References** |
|---|---|---|---|

| | | **vegetation ranges** | |
|---|---|---|---|
| Anthocyanin Reflectance Index-2 | $ARI2 = (R_{800} (1 / R_{550}) - (1 / R_{700})$ | 0 – 2; 0.001 – 0.1 | [35] |
| Carotenoid Reflectance Index-2 | $CRI2 = (1 / R_{515}) - ( 1 / R_{770})$ | 0 – 15; 1 – 11 | [35] |
| Structure Intensive Pigment Index | $SIPI = (R_{800} - R_{445}) / (R_{800} - R_{680})$ | 0 – 2; 0.8 – 1.8 | [36] |
| Flavonols Reflectance Index | $FRI = (1 / R_{410} - 1 / R_{460}) \times R_{800}$ | (– 0.2) – 0.8 Not studied | [37] |
| Greenness Index | $G = R_{554} / R_{677}$ | (– 1) – 1 | [38] |
| Greenness Index-2 | $G2 = R_{570} / R_{670}$ | (– 1) – 1; 0 – 1 | [39] |
| Redness Index | $R = R_{700} / R_{670}$ | (– 1) – 1 | [40] |
| Blue Index | $B = R_{450} / R_{490}$ | Not defined | [36] |
| Blue Green Pigment Index-2 | $BGI2 = R_{450} / R_{550}$ | (– 1) – 1 not precisely defined | [40] |
| Browning Reflectance Index-2 | $BRI2 = R_{450} / R_{690}$ | 0.2 – 0.8 | [40] |
| Lichtenthaler's Index-1 | $LIC1 = (R_{800} - R_{680}) / (R_{800} + R_{680})$ | 20 – 50 | [41] |
| Pigments Specific Simple Ratio-a | $PSSRa = R_{800} / R_{675}$ | (– 1) – 1; 0.2 – 0.8 | [42] |
| Pigments Specific Simple Ratio-b | $PSSRb = R_{800} / R_{650}$ | (– 1) – 1; 0.2 – 0.6 | [42] |
| Gitelson and Merzlyak Index | $GM1 = R_{750} / R_{550}$ | (– 1) – 1 | [38] |
| Gitelson and Merzlyak Index-2 | $GM2 = R_{750} / R_{700}$ | (-1) – 1; 0 – 1, | [38] |
| Zarco-Tejada Miller (RedEdge) Index | $ZMI = R_{750} / R_{710}$ | 0.4 – 1.2 | [43] |
| Normalized Difference Vegetation Index | $NDVI = (R_{800} - R_{680}) / (R_{800} - R_{680})$ | (– 1) – 1; 0.2 – 0.8 | [44] |
| Simple Ratio | $SR = R_{800} / R_{670}$ | (0 – ∞) not precisely defined. | [45] |
| Red-eye Vegetation Stress Index | $RVSI = (R_{714} + R_{752}) / (2 - R_{733})$ | 0 – 1 | [46] |
| Photochemical Reflectance Index | $PRI = (R_{531} - R_{570}) / (R_{531} + R_{570})$ | (– 1) – 1; (– 0.2) – 0.2 | [47] |
| Water Band Index | $WBI = R_{900} / R_{970}$ | 0.8 – 1.2; 0 – 1 | [48] |
| Photochemical Reflectance Index-515 | $PRI515 = (R_{515} - R_{531}) / (R_{515} + R_{531})$ | (– 1) – 1 | [49] |
| Specific disease index for individual study | $fD = I_{550} / (I_{550} + I_{690})$ | (– 1) – 1 | [50] |
| Healthy–index | $HI = ((R_{534} - R_{698}) / (R_{534} + R_{698})) - (\frac{1}{2} \times R_{704})$ | 0.2 – 1.2 | [24] |
| Plant Senescence Reflectance Index | $PSRI = (W_{680} - W_{500}) / W_{570}$ | (– 1) – 1; (– 0.1) – 0.2 | [51] |
| Vogelmann Red Edge Index | $VREI1 = R_{740} / R_{720}$ | 0 – 20; 4 – 8 | [52] |
| Red Edge Normalized Difference Vegetation Index | $RENDVI = (R_{750} - R_{705}) / (R_{750} + R_{705})$ | (– 1) – 1; 0.2 – 0.9 | [53,54] |
| Modified Red Edge Simple Ratio Index | $MRESRI = (R_{705} - R_{445}) / (R_{705} / R_{455})$ | 0 – 30; 2 – 8 | [54,55] |
